# Effect of fat emulsion administration on blood coagulation in terminal lung cancer patients

**DOI:** 10.20407/fmj.2021-012

**Published:** 2022-07-22

**Authors:** Takaki Kanie, Akihiko Futamura, Tomohiro Mizuno, Shigeki Yamada, Masanobu Usui

**Affiliations:** 1 Department of Pharmacy, Fujita Health University Hospital, Toyoake, Aichi, Japan; 2 Department of Pharmacy, Fujita Health University Nanakuri Memorial Hospital, Tsu, Mie, Japan; 3 Department of Palliative Medicine, Fujita Health University Hospital, Toyoake, Aichi, Japan

**Keywords:** Fat emulsion, Terminal lung cancer, Coagulation-fibrinolytic system, Prothrombin time, Activated partial thromboplastin time

## Abstract

**Objectives:**

Patients with cancer, especially those with lung cancer, are at high risk of developing thrombosis. Intralipos^®^ infusion 20% is contraindicated for thrombosis, and there is no consensus on whether it can be safely used in cases of advanced cancer. We conducted a retrospective observational study to elucidate the impact of fat emulsion administration on blood coagulation in patients with terminal lung cancer.

**Methods:**

The subjects were patients with terminal lung cancer in the Department of Surgery and Palliative Medicine, Fujita Health University Nanakuri Memorial Hospital between January 2016 and December 2019. We compared changes in their blood coagulation profile before hospitalization and one month later.

**Results:**

There were a total of 213 patients with lung cancer—139 who were administered fat emulsion and 74 who were not—with no significant differences in baseline characteristics. In the fat emulsion administration group (n=27), the prothrombin time-international normalized ratio (PT-INR) and activated partial thromboplastin time (APTT), respectively, were 1.17±0.26 (mean±standard deviation) and 30.5±5.0 s at hospitalization and 1.16±0.12 and 31.2±4.2 s one month later with no significant differences. In the non-administration group (n=6), the PT-INR and APTT, respectively, were 1.44±0.43 and 30.6±5.2 s before hospitalization and 1.28±0.18 and 33.0±7.5 s one month later with no significant differences.

**Conclusions:**

We did not identify any changes in PT-INR and APTT after fat emulsion administration in patients with terminal lung cancer. There were also no new cases of thrombosis, suggesting that fat emulsions were administered safely in patients with terminal lung cancer.

## Introduction

Most patients with cancer, especially those with terminal cancer, are malnourished. These patients need proper nutritional management to improve their quality of life (QOL) and prolong prognosis of patients. In recent years, nutritional management for patients with terminal stage, has become largely systematized.^1^ It is not uncommon for patients with terminal cancer to receive combined parenteral nutrition due to decreased oral intake. Parenteral nutrition needs lipids in addition to glucose and amino acids.

Fat emulsion is contraindicated in patients with thrombosis, and there is yet to be a consensus on whether it can be safely used in patients with advanced cancer,^2^ in whom blood coagulation is enhanced and thrombus formation is likely to occur.^3^ However, there is a contradictory view that the administration of fat emulsion increases the amount and rate of thrombin production but does not change the partial thromboplastin time.^4^ Moreover, a study on fat emulsion administration in patients with advance esophageal cancer showed no effect on primary hemostasis, platelet adhesion, or fibrin thrombus formation.^5^

To elucidate the impact of fat emulsion administration on blood coagulation in patients with terminal lung cancer, we investigated changes in blood coagulation capacity in patients with terminal lung cancer, who have a high risk of developing venous thrombosis,^6^—and investigated their risk for thrombosis.

## Methods

The subjects of this study were patients with lung cancer who had been admitted to the Department of Surgery and Palliative Medicine, Fujita Health University Nanakuri Memorial Hospital between January 2016 and December 2019. We compared changes in their blood coagulation at hospitalization and at one month after hospitalization. As selection criteria, we included patients with less than 3 months left to live, referring to the definition of terminal stage disease provided by the Japan Medical Association, as well as patients who had received Intralipos^®^ Injection 20% (Otsuka Pharmaceutical Factory, Inc.) at hospitalization and did not discontinue its use until one month later. The guidelines for infusion treatment for patients with terminal cancer^7^ state that fat emulsion administration may be considered for patients who have difficulty intaking meals. Thus, fat emulsion administration was decided at the discretion of the physician rather than by randomization. Exclusion criteria included patients who had contradictions for receiving Intralipos^®^ Injection 20%, patients with a prognosis exceeding 3 months, patients for whom hematological findings could not be tracked, and patients who developed infection.

This retrospective observational study compared the fat emulsion administration group and non-administration group. The safety parameters evaluated were prothrombin time-international normalized ratio (PT-INR), activated partial thromboplastin time (APTT), triglyceride (TG), aspartate transaminase (AST), alanine transaminase (ALT), white blood cell (WBC), red blood cell (RBC), and platelet (PLT). The efficacy parameters evaluated included albumin (ALB), transthyretin (TTR), and total cholesterol (TC). Comparisons were made between these parameters at hospitalization and at one month after hospitalization.

Fisher’s exact test was used to compare nominal variables between the groups. To compare non-normally distributed continuous variables, the Wilcoxon signed-rank sum test or the Mann-Whitney U test was performed. The t-test or paired t-test were performed for the comparison of normally distributed continuous variables. P-values <0.05 were considered statistically significant. Each parameter, unless otherwise specifically mentioned, was shown in terms of the mean±standard deviation. EZR version 1.41, a statistical analysis software that extends the functions of R and R Commander, was used for all statistical analyses. EZR is distributed free of charge on the website of the Department of Hematology, Jichi Medical University Saitama Medical Center.^8^

This study was conducted with approval from the Medical Ethics Committee of Fujita Health University (Reference No.: HM20-542). This study was conducted in accordance with the recommendations outlined in the Declaration of Helsinki (latest revision). In addition, information related to the implementation of this study was disclosed to the subjects based on the “Ethical Guidelines for Medical and Health Research Involving Human Subjects,” with an option for them to opt-out of the study.

## Results

**Figure 1:**
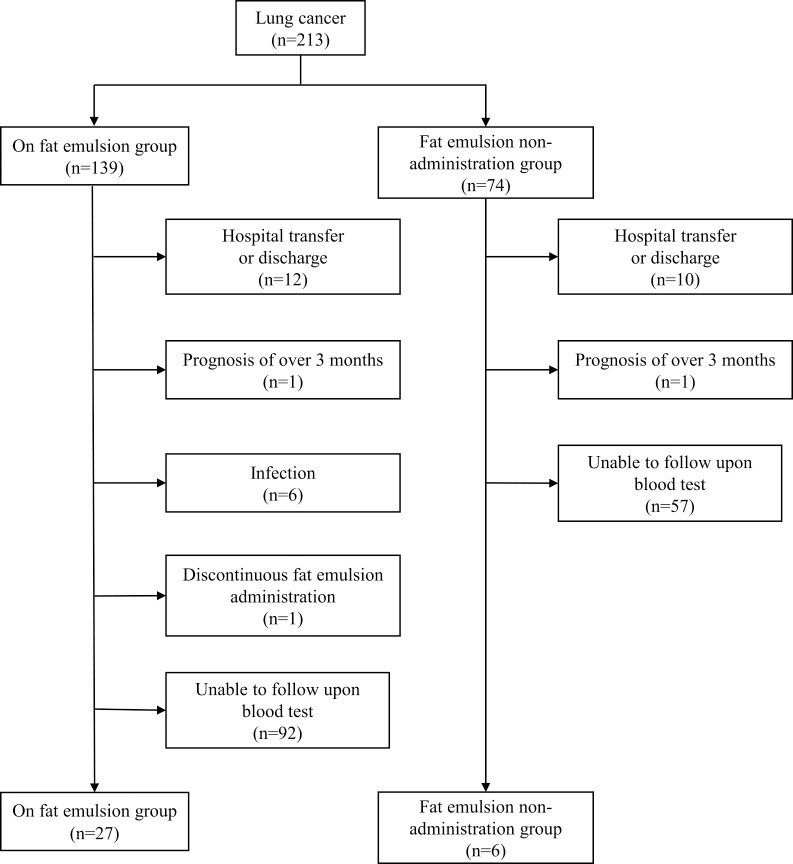
Flowchart of patient selection.

Figure 1 shows the flowchart of patient selection for this study. There were a total of 213 patients with lung cancer who were admitted to our hospital between January 2016 and December 2019, of whom 139 patients were administered Intralipos^®^ emulsion 20% infusion (fat emulsion administration group) and 74 patients were not (non-administration group). Baseline characteristics of the two groups are shown in Tables 1 and 2. Aside from ALB levels, no significant differences were observed in the background parameters of the two groups. After excluding patients according to the exclusion criteria, those who were transferred or discharged over the course of the study period, and those who had stopped receiving Intralipos^®^ emulsion 20% for infusion while being hospitalized, the final analysis set comprised 33 patients—27 in the fat emulsion administration group and 6 in the non-administration group.

There were no significant differences in background characteristics between the analyzed patients in the fat emulsion administration group and those in the non-administration group (Tables 3 and 4). However, there was a large difference in the ratio of female patients (40% in the fat emulsion administration group vs 17% in the non-administration group), and the median survival was longer in the non-administration group by approximately 14 days. Furthermore, there were four patients with performance status of 4 in the fat emulsion administration group and none in the non-administration group, suggesting a trend towards multi-organ metastasis in the fat emulsion administration group.

In the fat emulsion group, 25 patients received 100 mL of Intralipos^®^ emulsion 20% for infusion, and two patients received 250 mL of the same. The amount of fat emulsion preparation administered was 1 bag/day at a rate of 20 mL/hour (100 mL: 5 h; 250 mL: 12.5 h), and the mean administration duration was 31±9 days.

The mean energy of the administered dose in the fat emulsion administration group and the non-administration group on admission was 900±280 kcal and 550±220 kcal (p<0.05), respectively, and 980±360 kcal and 460±450 kcal (p<0.05), respectively, after one month. There were no significant differences in the energy of the administered dose upon admission and one month later in either group (fat emulsion administration group, p=0.194; non-administration group, p=0.525).

**Figure 2:**
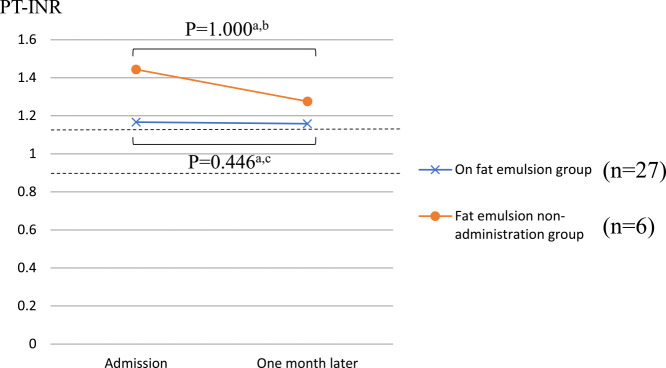
Comparison of PT-INR between the two groups. The values of PT-INR (SD: standard deviation) in the fat emulsion group were 1.17±0.26 (at admission) and 1.16±0.12 (one month later). The values of PT-INR (SD) in the fat emulsion non-administration group were 1.44±0.43 (at admission) and 1.28±0.18 (one month later). ^a^ Wilcoxon signed-rank test, ^b^ 1-β=0.174, ^c^ 1-β=0.0405. PT-INR, prothrombin time-international normalized ratio.

**Figure 3:**
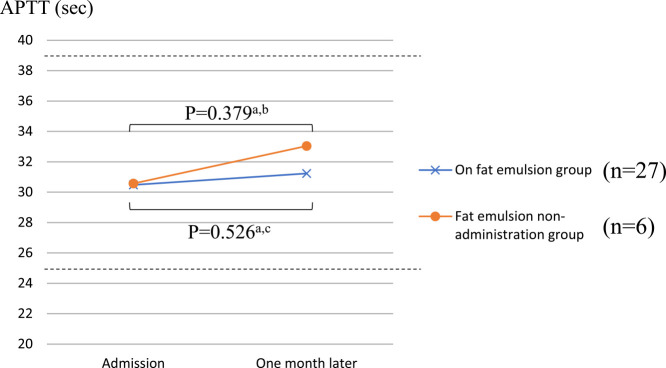
Comparison of APTT between the two groups. The values of APTT (SD) in the fat emulsion group were 30.5±5.0 (at admission) and 31.2±4.2 (one month later). The values of APTT (SD) in the fat emulsion non-administration group were 30.6±5.2 (at admission) and 33.0±7.5 (one month later). ^a^ paired t test, ^b^ 1-β=0.121, ^c^ 1-β=0.132. APTT, activated partial thromboplastin time.

Figures 2 and 3 show the PT-INR and APTT, respectively, in the two groups upon admission and one month later. In the administration group, the PT-INR was 1.17±0.26 at admission and 1.16±0.12 one month later, whereas in the non-administration group, the PT-INR was 1.44±0.43 at admission and 1.28±0.18 one month later. In the administration group, the APTT was 30.5±5.0 s at admission and 31.2±4.2 s one month later, whereas in the non-administration group, the APTT was 30.6±5.2 s at admission and 33.0±7.5 s one month later. There were no significant differences in PT-INR and APTT at admission and one month later in either group.

Table 5 shows the hematological findings in the fat emulsion administration group at admission and one month later. There were no significant differences in TG, ALT, and PLT before and after fat emulsion administration. At admission and one month later, ALB was 3.1±0.6 g/dL and 2.5±0.5 g/dL, respectively; TTR was 16.5±9.0 mg/dL and 12.7±6.7 mg/dL, respectively; TC was 182.5±41.4 mg/dL and 158.2±46.4 mg/dL, respectively; and RBC was 4.01±0.66 (×10^3^/μg) and 3.68±0.73 (×10^3^/μg), respectively; these were significantly lower at admission than at one month later (p<0.05). On the other hand, at admission and one month later, C-reactive protein (CRP) was 4.19±4.76 mg/dL and 7.40±5.77 mg/dL, respectively; AST was 27.9±13.0 IU/L and 36.8±23.7 IU/L, respectively; and WBC was 9.1±4.2×10^3^/μg and 11.5±5.5×10^3^/μg, respectively, showing a significant increase (p<0.05).

There were no significant differences in hematological findings in the non-administration group at admission and one month later (Table 6).

## Discussion

We investigated the PT-INR and APTT before and after fat emulsion administration in patients with terminal lung cancer to study the effect of fat emulsion administration on blood coagulation in such patients.

In the present study, patients with lung cancer who were admitted to the Department of Surgery and Palliative Medicine, Fujita Health University Nanakuri Memorial Hospital between January 2016 and December 2019. There were no significant differences in background parameters between patients in the fat emulsion administration group and the non-administration group. Although there were no differences in background parameters between the 33 patients analyzed, Survival time after exclusion was prolonged than before exclusion. This was because patients with a short survival who were not followed-up one month later were excluded.

There was no significant difference in PT-INR or APTT at admission and one month after admission in either group, suggesting that the administration of Intralipos^®^ Injection 20% does not affect blood coagulation in patients with terminal lung cancer. However, this might have occurred due to the small detection power or potential secondary errors. Regarding safety parameters, no significant difference was observed in the TG of either group, suggesting that fat emulsion administration has little impact on lipid metabolism. Motton et al. investigated the effect of fat emulsion on blood coagulation in 26 patients with advanced esophageal cancer receiving total parenteral nutrition (TPN) and reported that no changes in coagulation system and platelet adhesion were observed.^5^ Although Reid reported that APTT and PT suggested enhanced coagulation trends after fat emulsion administration, that the change was mild and not clinically problematic.^4^ Tappy et al. reported that high-fat-containing (70% of total energy) TPN could be safely administered for 5 days without TG accumulation.^9^ Our results support these reports, suggesting that fat emulsion preparations can be safely used for patients with terminal lung cancer without affecting blood coagulation or lipid metabolism.

In a moderately invasive gastrointestinal surgery, Haji et al. reported that fluid therapy with 30% of total energy as lipids improved protein metabolism compared to equal-energy fat-free infusions (glucose+amino acids).^10^ Koshi et al. administered a fat emulsion by peripheral parenteral nutrition at a fat ratio of 60.9% to the amount of energy required for a patient fasting for a gastrointestinal disease for about 7 days; there were no significant changes in TG and TC before and after the administration.^11^ Although TTR, ALB, and TC as the efficacy parameters decreased significantly in the fat emulsion administration group, these parameters did not vary significantly in the non-administration group. The actual amount of emulsion administered in terms of energy at admission was significantly higher in the fat emulsion administration group than in the non-administration group. Furthermore, the CRP one month later was significantly higher in the former. These suggest that catabolism was enhanced, and active nutritional management was applied in the fat emulsion administration group. The significantly higher level of AST and the inclusion of patients with liver metastases in the fat emulsion administration group might have also contributed to the decline in TTR, ALB, and TC.

Limitations of this study include its retrospective design, small sample size, targeting of patients with terminal cancer, and inclusion of patients capable of oral intake. In addition, fat emulsion was administered to patients at the discretion of the physician. Patients who were not selected for fat emulsion administration might have had irreversible cachexia and a prognosis of several days. Therefore, it is possible that patients with low CRP, which is also used to predict prognosis, were selected. The level of CRP is correlated with that of IL-6.^12^ Because IL-6 causes vascular endothelial damage and leads to thrombus formation,^13^ patients with low IL-6 (i.e., low CRP) may be less likely to develop hypercoagulation than patients with high CRP. Because no hypercoagulation was observed regardless of fat emulsion administration in this study, the above factors had little impact on blood coagulation. This study suggests that administering fat emulsion preparations in patients with terminal lung cancer is safe. However, statistically significant differences were not noted, likely due to the small statistical detection power and potential secondary errors. Further research should be conducted with a larger sample size to guarantee statistical detection.

As for efficacy, contrary to our prediction, fat emulsion administration reduced TC. TC has recently been regarded as an important nutritional and prognostic index, especially in patients with terminal cancer.^14^ As subjects in this study were patients with terminal cancer having a median survival period of 54 days and facing imminent death, fat emulsion might not have been effectively used in the body, and our results may reflect this deterioration in general condition. As the sample size was small, similar studies with patients having a prognosis of around 6 months should be conducted. In addition, we analyzed patients who were receiving parenteral nutrition in addition to oral intake, and it is possible that their metabolic dynamics were different from those who received parenteral nutrition alone. we could not elucidate the association between these factors and the reduction of TC.

We excluded patients with a vital prognosis of ≥3 months and patients who developed infections. The large effect of inflammation on nutritional status makes it difficult to evaluate the impact of fat emulsion administration. Therefore, the results of this study are applicable to patients with terminal lung cancer who have a relatively good prognosis and do not show a strong inflammatory response. In fact, when excluded patients were compared to included patients, the included patients who had received fat emulsion showed significantly reduced APTT, high ALB and TTR, and low CRP. This suggests that the included patients had little inflammatory response and a favorable nutritional status, thereby supporting the above hypothesis.

There were several limitations due to the limited number of subjects, and there was insufficient consideration regarding a causal relationship. In further study, it is necessary to increase the sample size and conduct studies that consider energy charge and the effect of combining parenteral nutrition with oral intake.

## Figures and Tables

**Table1:** Baseline patient characteristics (physical examination findings)

		On fat emulsion group (n=139)	Fat emulsion non-administration group (n=74)	p-value
Age (years)		77.6±9.2	77.7±9.9	0.752^a^
Sex (female)		41	20	0.752^b^
Height (cm)		159.6±10.1	158.9±10.3	0.666^a^
Weight (kg)		48.6±10.3	49.7±12.0	0.724^a^
BMI (kg/m^2^)		19.0±3.3	19.6±4.2	0.664^a^
Lung cancer				
	Small-cell	14	7	0.716^b^
	Non-small cell	82	40	
	Unknown	43	27	
Survival time				
	Median (day)	25	16.5	0.135^a^
Performance status				
	2	2	1	0.203^b^
	3	44	32	
	4	92	41	
Metastasis				
	Liver	27	18	0.766^b^
	Bone	44	19	
	Peritoneum	8	4	
	Brain	47	19	
	Lymph node	30	16	

Values are expressed as the mean±standard deviation. ^a^ Mann–Whitney U test, ^b^ Fisher’s exact test. BMI, body mass index.

**Table2:** Baseline patient characteristics (blood investigation findings)

	Normal range	On fat emulsion group (n=139)	Fat emulsion non-administration group (n=74)	p-value
PT-INR	0.86–1.09	1.25±0.4	1.27±0.3	0.514^a^
APTT (sec)	24–39	32.9±6.5	33.0±7.1	0.921^a^
ALB (g/dL)	4.1–5.1	2.8±0.7	2.6±0.6	0.017^b^
TTR (mg/dL)	22–40	13.4±7.5	12.0±6.6	0.296^a^
CRP (mg/dL)	0–0.14	6.5±7.3	7.4±7.4	0.245^a^
TC (mg/dL)	124–222	173.2±51.3	168.9±44.5	0.6^a^
TG (mg/dL)	30–149	115.5±54.3	121.8±57.8	0.48^a^
AST (IU/L)	13–20	43.1±65.5	46.3±55.9	0.877^a^
ALT (IU/L)	10–42	32.5±43.3	32.6±42.4	0.688^a^

Values are expressed as the mean±standard deviation. ^a^ Mann–Whitney U test, ^b^ t-test. PT-INR, prothrombin time-international normalized ratio; APTT, activated partial thromboplastin time; ALB, albumin; TTR, transthyretin; CRP, C-reactive protein; TC, total cholesterol; TG, triglyceride; AST, aspartate transaminase; ALT, alanine transaminase.

**Table3:** Underlying medical conditions and characteristics of the analyzed patients (physical examination findings)

		On fat emulsion group (n=27)	Fat emulsion non-administration group (n=6)	p-value
Age (years)		77.0±8.5	76.4±4.2	0.907^a^
Sex (female)		11	1	0.379^b^
Height (cm)		159.1±9.4	160.0±10.3	0.657^a^
Weight (kg)		47.7±10.7	48.9±11.9	0.815^a^
BMI (kg/m^2^)		18.1±4.9	18.9±3.0	0.640^a^
Lung cancer				
	Small-cell	2	1	0.466^b^
	Non-small cell	19	3	
	Unknown	6	2	
Survival time				
	Median (day)	54	67.5	0.207^a^
Performance status				
	2	2	1	0.264^b^
	3	16	5	
	4	9	0	
Metastasis				
	Liver	8	0	0.463^b^
	Bone	8	0	
	Peritoneum	2	1	
	Brain	12	1	
	Lymph node	9	1	

Values are expressed as the mean±standard deviation. ^a^ Mann–Whitney U test, ^b^ Fisher’s exact test. BMI, body mass index.

**Table4:** Underlying medical conditions and characteristics of the analyzed patients (blood investigation findings)

	Normal range	On fat emulsion group (n=27)	Fat emulsion non-administration group (n=6)	p-value
PT-INR	0.86–1.09	1.17±0.26	1.44±0.43	0.0161^a^
APTT (sec)	24–39	30.5±5.0	30.6±5.2	0.779^a^
ALB (g/dL)	4.1–5.1	3.1±0.6	2.8±0.3	0.23^b^
TTR (mg/dL)	22–40	16.5±9.0	17.5±8.6	0.691^a^
CRP (mg/dL)	0–0.14	4.19±4.76	3.98±5.05	0.815^a^
TC (mg/dL)	124–222	182.5±41.4	162.5±38.1	0.287^b^
TG (mg/dL)	30–149	118.4±43.2	105.2±41.4	0.726^a^
AST (IU/L)	13–20	27.9±13.0	33.7±20.3	0.513^a^
ALT (IU/L)	10–42	26.1±24.6	27.8±23.9	0.87^a^
WBC (×10^3^/μg)	3.3–8.6	9. 1±4.2	7.8±3.0	0.469^a^
RBC (×10^6^/μg)	3.86–4.92	4.01±0.66	3.68±0.61	0.268^b^
PLT (×10^4^/μg)	15.8–34.8	31.2±12.4	25.1±6.8	0.259^b^

Values are expressed as the mean±standard deviation. ^a^ Mann–Whitney U test, ^b^ t-test. PT-INR, prothrombin time-international normalized ratio; APTT, activated partial thromboplastin time; ALB, albumin; TTR, transthyretin; CRP, C-reactive protein; TC, total cholesterol; TG, triglyceride; AST, aspartate transaminase; ALT, alanine transaminase; WBC, white blood cell; RBC, red blood cell; PLT, platelet.

**Table5:** Impact on blood investigation findings in the fat emulsion administration group (n=27)

	Normal range	Admission	One month later	p-value
ALB (g/dL)	4.1–5.1	3.1±0.6	2.5±0.5	<0.05^b^
TTR (mg/dL)	22–40	16.5±9.0	12.7±6.7	<0.05^a^
CRP (mg/dL)	0–0.14	4.19±4.76	7.40±5.77	<0.05^a^
TC (mg/dL)	124–222	182.5±41.4	158.2±46.4	<0.05^b^
TG (mg/dL)	30–149	118.4±43.2	113.5±54.7	0.602^b^
AST (IU/L)	13–20	27.9±13.0	36.8±23.7	<0.05^a^
ALT (IU/L)	10–42	26.1±24.6	30.8±22.4	0.115^a^
WBC (×10^3^/μg)	3.3–8.6	9. 1±4.2	11.5±5.5	<0.05^a^
RBC (×10^6^/μg)	3.86–4.92	4.01±0.66	3.68±0.73	<0.05^b^
PLT (×10^4^/μg)	15.8–34.8	31.2±12.4	30.4±12.1	0.739^b^

Values are expressed as the mean±standard deviation. ^a^ Wilcoxon signed-rank test, ^b^ paired t-test. ALB, albumin; TTR, transthyretin; CRP, C-reactive protein; TC, total cholesterol; TG, triglyceride; AST, aspartate transaminase; ALT, alanine transaminase; WBC, white blood cell; RBC, red blood cell; PLT, platelet.

**Table6:** Impact on blood investigation findings in the non-administration group (n=6)

	Normal range	Admission	One month later	p-value
ALB (g/dL)	4.1–5.1	2.8±0.3	2.6±0.6	0.235^b^
TTR (mg/dL)	22–40	17.5±8.6	14.6±10.9	0.128^b^
CRP (mg/dL)	0–0.14	3.98±5.05	5.95±4.31	0.172^b^
TC (mg/dL)	124–222	162.5±38.1	161.5±53.0	0.918^b^
TG (mg/dL)	30–149	105.2±41.4	102.3±20.2	0.777^b^
AST (IU/L)	13–20	33.7±20.3	22.2±6.0	0.438^a^
ALT (IU/L)	10–42	27.8±23.9	12.5±6.6	0.136^a^
WBC (×10^3^/μg)	3.3–8.6	7.8±3.0	7.3±1.1	0.662^b^
RBC (×10^6^/μg)	3.86–4.92	3.68±0.61	3.78±0.75	0.435^b^
PLT (×10^4^/μg)	15.8–34.8	25.1±6.8	26.6±10.7	0.508^b^

Values are expressed as the mean±standard deviation. ^a^ Wilcoxon signed-rank test, ^b^ paired t-test. ALB, albumin; TTR, transthyretin; CRP, C-reactive protein; TC, total cholesterol; TG, triglyceride; AST, aspartate transaminase; ALT, alanine transaminase; WBC, white blood cell; RBC, red blood cell; PLT, platelet.

## Supplementary Material

PDF-Japanese
