# Supplementary material for: Effect of fat emulsion administration on blood coagulation in terminal lung cancer patients
Source: Fujita Med J. 2022 Jul 22;9(2):73–9. doi: 10.20407/fmj.2021-012 (PMC10206901; doi:10.20407/fmj.2021-012)
Supplement: Supplementary file 1 — PDF-Japanese [file fmj-9-073-s001.pdf]

Original article

論文表題：終末期肺がん患者の血液凝固に及ぼす脂肪乳剤投与の影響

著者

Takaki Kanie[1*], BPharm, Akihiko Futamura, PhD[2*], Tomohiro Mizuno, PhD[1], Shigeki Yamada, PhD[1], Masanobu Usui, MD, PhD[3]

1 Department of Pharmacy, Fujita Health University Hospital, Toyoake, Aichi, Japan

2 Department of Pharmacy, Fujita Health University Nanakuri Memorial Hospital, Tsu, Mie,Japan

3 Department of Palliative medicine, Fujita Health University Hospital, Tsu, Mie, Japan

*These authors contributed equally to this article

ランニングタイトル：終末期肺がん患者の血液凝固に及ぼす脂肪乳剤投与の影響

Corresponding author：

Akihiko Futamura, PhD

Department of Pharmacy, Fujita Health University Nanakuri Memorial Hospital, 424-1, Oodoricho, Tsu, Mie, 514-1295, Japan

E-mail：futamura@fujita-hu.ac.jp

Takaki Kanie

Department of Pharmacy, Fujita Health University Hospital, 1-98 Dengakugakubo, Kutsukake-cho, Toyoake, Aichi 470-1192, Japan

E-mail：takaki.kanie@fujita-hu.ac.jp　Tell：0562-93-2157

アブストラクト

目的

　がん患者、特に肺がん患者では血栓症のリスクが高いことが報告されている。イントラリポス®輸液20%は血栓症に禁忌とされ、進行がんの病態に安全に使用できるか統一した見解は明らかになっていない。血液凝固亢進している可能性のある終末期肺がん患者において、脂肪乳剤投与が凝固系に及ぼす影響を明らかにするべく後方視的観察研究を実施する。

方法

　対象は2016年1月から2019年12月までに藤田医科大学七栗記念病院外科・緩和ケア科に入院した肺がん患者とし、入院時および入院1カ月後における血液凝固系の変化を比較検討する。

結果

　肺がん患者は213名で投与群139名、非投与群74名であり背景に差はなかった。投与群のうち1か月後に評価できたのは27名で、プロトロンビン時間国際標準比（PT-INR）および活性化部分トロンボプラスチン時間（APTT）はそれぞれ入院時1.17±0.26（平均±標準偏差）、30.5±5.0秒、1カ月後1.16±0.12、31.2±4.2秒と有意差はなかった。非投与群では6名で、PT-INRおよびAPTTは入院時1.44±0.43、30.6±5.2秒、1カ月後1.28±0.18、33.0±7.5秒といずれも有意差はなかった。

結論

終末期肺がん患者において脂肪乳剤投与による PT-INR、APTT の変化を認めなかった。また、新たな血栓症発症も認めず、血液凝固亢進の可能性のある終末期肺がん患者への脂肪乳剤の投与は、安全に使用できると考えられたが、今後症例数を増やして詳細に調査する必要がある。

キーワード：脂肪乳剤、終末期、凝固線溶系、プロトロンビン時間、活性化部分トロンボプラスチン時間

序論

がん患者、特に終末期がん患者は、そのほとんどが栄養障害を有しており症状や生活の質（Quality Of Life）の改善、生命の延長を得るためには適切な栄養管理が必要となる。近年、終末期を含むがん患者に対する栄養管理が大きく体系づけられるようになってきた[1]。終末期がん患者は経口摂取量の減少から静脈栄養を併用することは珍しくない。静脈栄養を行う際には糖やアミノ酸の投与だけでなく、脂質の投与も必要となる。

一方、脂肪乳剤は血栓症患者などに禁忌とされており、血液凝固能が亢進し血栓を形成しやすくなる進行がんの病態[2]においては、安全に使用できるか未だ統一した見解は明らかになっていない。

脂肪乳剤が血栓症を有する患者に対し禁忌となった理由としては、脂肪乳剤を混和した血液検体においてトロンビンの生成量と速度の増加が認められたためとされている[3]。しかし、脂肪乳剤投与によりトロンビンの生成量および速度は増加するが部分トロンボプラスチン時間としては変化が見られないという否定的な意見がある[4]。また、ステージⅢおよびⅣの食道がん患者を対象にした研究が唯一あり[5]、一次止血、血小板粘着、フィブリン血栓形成に影響はないという結果であった[5]。

本研究では、静脈血栓症の危険性が高い肺がん[6]において終末期がん患者を対象に脂肪乳剤投与による血液凝固能の変動を調査し、血栓症の発症リスクについて検討し

た。

方法

　対象は 2016 年 1 月から 2019 年 12 月までに藤田医科大学七栗記念病院緩和ケア・外科に入院した肺がん患者とし、入院時および入院 1 カ月後における血液凝固系の変化を調査した。選択基準として、日本医師会の終末期の定義を参考に死亡前 3 か月以内の患者、かつイントラリポス®輸液 20%を入院時に投与開始され入院 1 カ月後まで中止のない患者を抽出した。脂肪乳剤の投与は終末期癌患者に対する輸液治療のガイドライン[7]で、食事摂取が困難な患者に対して投与を検討できるとされており、無作為割り付けではなく医師の裁量により投与が決定された。除外基準はイントラリポス®輸液 20%の禁忌に当てはまる患者、予後 3 か月を超える患者、血液検査所見を追跡できない患者、感染症を発症した患者とした。

　試験デザインは後方視的観察研究とし、脂肪乳剤の投与群と非投与群の 2 群間比較試験とした。安全性の評価項目は、プロトロンビン時間国際標準比（prothrombin time-international normalized ratio：PT-INR）、活性化部分トロンボプラスチン時間（activated partial thromboplastin time：APTT）、中性脂肪（triglyceride：TG）、アスパラギン酸アミノトランスフェラーゼ（aspartate transaminase：AST）、アラニンアミノトランスフェラーゼ（alanine transaminase：ALT）、白血球数（white blood cell：WBC）、赤血球数（red blood cell：RBC）、血小板数（platelet：PLT）そして、有効

性の評価には、アルブミン（albumin：ALB）、トランスサイレチン（transthyretin：TTR）、総コレステロール（total cholesterol：TC）とし、入院時と入院後 1 カ月の PT-INR、APTT、TG、AST、ALT、WBC、RBC、PLT、ALB、TTR、TC の変化を比較検討した。

　統計処理としては、群間比較において名義変数の比較には Fisher の正確確率検定を行う。連続変数の比較はデータの正規性と分散性を確認したうえで、非正規分布であれば Wilcoxon の符号付順位和検定または Mann-Whitney U 検定を、正規分布であれば t 検定または対応のある t 検定を行う。いずれも得られた p 値が 0.05 未満の場合に統計的有意差ありと判断する。また、各項目は特に記載がない限り平均値±標準偏差で示した。統計解析ソフトウェアは EZR ver1.41 を用いた。EZR は R および R コマンダーの機能を拡張した統計ソフトであり、自治医科大学附属さいたま医療センター血液内科のホームページで無償配布されている[8]。

　本研究は藤田医科大学の医学研究倫理審査委員会において承認を得たうえで実施した（受付番号：HM20-542）。なお、本研究は「人を対象とする医学系研究に関する倫理指針」に基づき、研究の実施についての情報を研究対象者に公開し、オプトアウトを実施するとともに、個人情報保護には十分配慮し、患者に関する情報は匿名化し取り扱った。

結果

対象となった患者を Figure 1 に示す。2016 年 1 月から 2019 年 12 月までの期間で入院した肺がん患者は 213 名であった。その内、イントラリポス®輸液 20%を投与した群（投与群）は 139 名、投与していない群（非投与群）は 74 名であった。両群の背景を Table 1,2 に示す。両群の背景は ALB 以外に差は見られなかった。除外基準に該当する患者を除いて条件を満たした最終的な解析対象患者は 33 名であり、経過中に転院または退院した患者、イントラリポス®輸液 20%が入院期間中に中止となった患者も除外とした。解析対象患者 33 名を対象に投与群 27 名、非投与群 6 名として検討した。

解析対象となった患者背景を Table 3,4 に示す。投与群と非投与群に差はなかった。しかし、女性の割合は投与群 40%、非投与群 17%と大きく違い、生存期間の中央値は非投与群の方が約 14 日間長い。また、performance status 4 の患者は投与群で 4 名、非投与群ではおらず、転移において投与群の方が複数臓器に転移している傾向にある。

投与群 27 名に投与された脂肪乳剤はイントラリポス®輸液 20%100mL が 25 名、250mL が 2 名だった。脂肪乳剤の投与量は 100mL および 250mL とも 1 袋/日、投与速度は 20mL/時（100mL：5hr、250mL：12.5hr)、投与期間は平均 31±9 日であった。

平均エネルギー実投与量は、入院時において投与群および非投与群でそれぞれ 900±280kcal、550±220kcal（$p<0.05$）であり、入院 1 カ月後では 980±360kcal、460

±450kcal（p<0.05）であった。また、入院時および入院 1 カ月後のエネルギー実投与量を比較すると投与群で p=0.194、非投与群で p=0.525 であり有意差はなかった。

Figure 2 に両群ごとの入院時と入院 1 カ月後の PT-INR を示す。投与群では入院時、入院 1 カ月後でそれぞれ 1.17±0.26、1.16±0.12、非投与群では 1.44±0.43、1.28±0.18 であった。同様に Figure 3 には APTT を示す。投与群ではそれぞれ 30.5±5.0 秒、31.2±4.2 秒、非投与群では 30.6±5.2 秒、33.0±7.5 秒だった。両群とも入院時と入院 1 カ月後の PT-INR および APTT に差は認められなかった。

Table 5 に投与群における入院時と入院 1 カ月後の血液検査所見を示す。TG、ALT、PLT では投与前後の検査値に有意差は認められなかった。ALB は入院時、入院 1 カ月後でそれぞれ 3.1±0.6g/dL、2.5±0.5g/dL、TTR は 16.5±9.0mg/dL、12.7±6.7mg/dL、TC は 182.5±41.4mg/dL、158.2±46.4mg/dL、RBC は 4.01±0.66(×10^3/μg)、3.68±0.73(×10^3/μg)であり有意に低下していた(p<0.05)。CRP は 4.19±4.76mg/dL、7.40±5.77mg/dL、AST は 27.9±13.0IU/L、36.8±23.7IU/L、WBC は 9. 1±4.2×10^3/μg、11.5±5.5×10^3/μg であり有意に増加していた（p<0.05)。

Table 6 に非投与群における入院時と入院 1 カ月後の血液検査所見を示す。TTR、ALB、CRP、TC、TG、AST、ALT 、WBC、RBC、PLT のいずれも有意差を認めなかった。

考察

今回我々は、終末期がん患者の脂肪乳剤投与による血液凝固への影響について肺がん患者を対象に脂肪乳剤投与前後の PT-INR、APTT を調査した。

2016 年 1 月から 2019 年 12 月までに藤田医科大学七栗記念病院緩和ケア・外科に入院した肺がん患者 213 名を対象にしており、イントラリポス®輸液 20%投与群と非投与群の両群間で背景に差は見られなかった。解析対象者の 33 名の背景でも差は見られなかったが、生存期間が除外前と除外後で延びている。これは、血液検査所見が 1 カ月後まで追えない生存期間の短い患者が除外されたためと考えられる。

イントラリポス®輸液 20%投与群と非投与群の両群とも入院時と入院 1 カ月後の PT-INR、APTT において有意差は認められなかったことから、終末期肺がん患者ではイントラリポス®輸液 20%の投与により凝固系に影響は及ぼさないことが示唆された。しかし、有意差が認められなかったのは、検出力が小さく第二の過誤が生じている可能性もある。安全性の評価では両群の TG において有意差は認められず、脂質代謝への影響も少ないと考えられた。Motton らは、中心静脈栄養（Total Parenteral Nutrition：TPN）を受けているステージⅢおよびⅣの食道がん症例 26 例において脂肪乳剤の血液凝固能に与える影響を検討しており、脂肪乳剤による凝固系や血小板粘着能への変化は認めなかったとしている [5]。また、Reid は脂肪乳剤の投与により APTT、PT は凝固亢進を認めるが軽微であり、臨床的には問題ないと報告している [4]。また、

Tappy らは TPN で総エネルギーのうち 70%を脂質とした高脂肪含有 TPN を 5 日間投与した結果、TG の蓄積なく安全に投与できたと報告している[9]。今回はこれらの報告と同様な結果となり、終末期肺がん患者に脂肪乳剤を投与しても血液凝固および脂質代謝に影響なく安全に使用できることが示唆された。

土師らは中程度の侵襲がある消化器外科手術において、総エネルギーのうち 30%を脂質とした輸液療法は、等エネルギーの無脂肪輸液（糖質＋アミノ酸）と比べて蛋白代謝を改善したと報告している[10]。合志らは、消化器科疾患により絶食で、末梢静脈栄養が 7 日間程度必要と診断された症例に対し末梢静脈栄養で脂肪乳剤を必要エネルギー量に対する脂肪比率 60.9%で投与したところ、TG、TC は開始前と終了時で有意な変化はなかったと報告している[11]。今回、有効性の評価項目のうち TTR、ALB、TC は投与群で有意に減少し、非投与群では有意差を認めなかったとする予想に反した結果となった。この点について、両群の入院時のエネルギー実投与量を検討したところ、投与群が非投与群に比べて有意に高値であったことや投与群における 1 カ月後の CRP が非投与群に比べて有意に高値となっていたことから、何らかの要因で投与群では異化が亢進し栄養管理を強化していたことが考えられた。加えて、AST が投与群で有意に高値であったこと、投与群にのみ肝転移の患者が含まれていたことも TTR、ALB、TC が低下した一因かもしれない。

本研究の限界点として、後ろ向き研究であること、症例数が少ないこと、終末期が

ん患者であること、経口摂取している症例も含んでいることが挙げられる。また、本研究では医師の裁量により脂肪乳剤が投与された。脂肪乳剤投与が選択されなかった患者背景として、不可逆的悪液質の状態であること、予後が数日であることが考えられる。そのため、予後予測にも用いられる CRP の低い患者が選択された可能性がある。CRP が高値であると IL-6 も相関して高値となる[12]。IL-6 は血管内皮障害を引き起こし血栓形成へつながる[13]ため、IL-6 が低く、すなわち CRP が低い患者では、CRP が高い患者に比して、凝固系が亢進しにくい可能性がある。ただし、本研究では、脂肪乳剤投与の有無に関わらず、凝固系の亢進が認められなかったため、上記因子の影響は軽微であったと考えられる。今回終末期肺がん患者への脂肪乳剤投与における安全性は示唆された。しかし、統計学的有意差が認められなかった要因として、統計学的検出力が小さく、第二の過誤が生じているとも考えられる。今後、統計学的検出力を担保したより大きなサンプル数での研究が必要である。

　有効性に関しては、予想に反し脂肪乳剤を投与することで総コレステロールが低下していた。総コレステロールは最近栄養指標として重要視されており特に終末期がん患者においては予後の指標としても報告されている[14]。今回の対象が生存期間の中央値 54 日と死が差し迫った終末期がん患者であるため、脂肪乳剤が十分に体内で有効利用されなかった可能性もあり、全身状態の悪化を反映した結果であることも考えられる。また、症例数が投与群で 27 名、非投与群で 6 名と非投与群で少ないため、半

年程度の予後が見込める症例を加えて検討が必要であると考えられた。他に、経口摂取に加え静脈栄養を併用している患者も対象としており静脈栄養のみの患者と代謝動態が異なることも考えられる。今回はそれらの影響を検討することができなかった。

本研究では、予後 3 か月以上の患者および感染症を発症した患者は対象から除外した。この理由として、炎症反応による栄養状態への影響が極めて大きく、脂肪乳剤等による影響を評価できないためである。そのため、本研究の結果は、終末期肺がん患者の中でも予後が比較的良好かつ強い炎症反応を示していない患者にて、適応可能である。実際、除外患者群と対象患者群を比較したところ、脂肪乳剤が投与された対象患者群では、APTT が有意に短縮し ALB、TTR は高く、CRP が低かった。すなわち、対象患者では炎症反応が少なく栄養状態が良かったことが示唆され、上記仮説を支持する結果であった。

本研究では、研究対象患者数によりいくつかの限界があり因果関係について、十分な検討に至らなかった。今後は症例数を増やし、エネルギー充足率や静脈栄養の経口摂取との併用による影響を考慮した検討が必要と考える。

利益相反

本論文に関して，全ての著者に開示すべき利益相反関連事項はない。

引用文献

1 Higashiguchi T. Energy Metabolism and Management of Terminal Cancer Patients. Jomyaku Keicho Eiyo 2009; 24:1071-5 (in Japanese).

2 Madoiwa S. Why is venous thrombo-embolism a frequent complication in cancer patients?. Heart View 2018; 22: 130-6 (in Japanese).

3 Amris CJ, Brockner J, Larsen V. Changes in the coagulability of blood during the infusion of intralipid. Acta Chir Scand Suppl 1964; 325:70-4.

4 Reid DJ. Metabolic and blood coagulation changes during intravenous fat infusions. Ann R Coll Surg Engl 1968;42:322-36.

5 Motton G, Ricci F, Guglielmi A, Olivieri D, Cordiano C. Fat infusion and blood coagulation in patients undergoing surgery for esophageal cancer. The Ital J of Surg Sci 1984; 14: 271-4.

6 Horsted F, West J, Grainge MJ. Risk of venous thromboembolism in patients with cancer: a systematic review and meta-analysis. PLoS Med 2012; 9: e1001275.

7 Japanese Society for Palliative Medicine. Syuumatuki gannkannja ni taisuru yuekitiryou no guidline (Guidelines of Infusion Treatment for Terminal Cancer Patients). Japanese Society for Palliative Medicine; 2007: 41-42 (in Japanese).

8 Kanda Y. Investigation of the freely available easy-to-use software 'EZR' for medical statistics. Bone Marrow Transplant 2013; 48: 452-8.

9 Tappy L, Schwarz JM, Schneiter P, Cayeux C, Revelly JP, Fagerquist CK, Jéquier E, Chioléro R. Effects of isoenergetic glucose-based or lipid-based parenteral nutrition on glucose metabolism, de novo lipogenesis, and respiratory gas exchanges in critically ill patients. Crit Care Med 1998; 26: 860-7.

10 Haji S, Nomura H, Ohyanagi H. Effect of nutrient substrates for protein metabolism under surgical stress. Japanese Journal of Nutritional Assessment 2000; 17: 47-53 (in Japanese).

11 Goshi S, Kamuro T, Suzuki N, Takei S. The Safety when administered a high proportion of lipids emulsions in peripheral parenteral nutrition. The Journal of Japanese Society for Parenteral and Enteral Nutrition 2015; 30:1285-92 (in Japanese).

12 Blay JY, Negrier S, Combaret V, Attali S, Goillot E, Merrouche Y, Mercatello A, Ravault A, Tourani JM, Moskovtchenko JF, Thierry P, Marie F Serum level of interleukin 6 as a prognosis factor in metastatic renal cell carcinoma. Cancer Res 1992; 52: 3317-22.

13 Seiji Madoiwa Malignancy and Thrombosis. The Medical Frontline 2010; 65: 1137-46.

14 Ignacio de Ulíbarri J, González-Madroño A, de Villar NG, González P, González B, Mancha A, Rodríguez F, Fernández G. CONUT: A tool for controlling nutritional status. First validation in a hospital population. Nutr Hosp 2005; 20: 38-45.

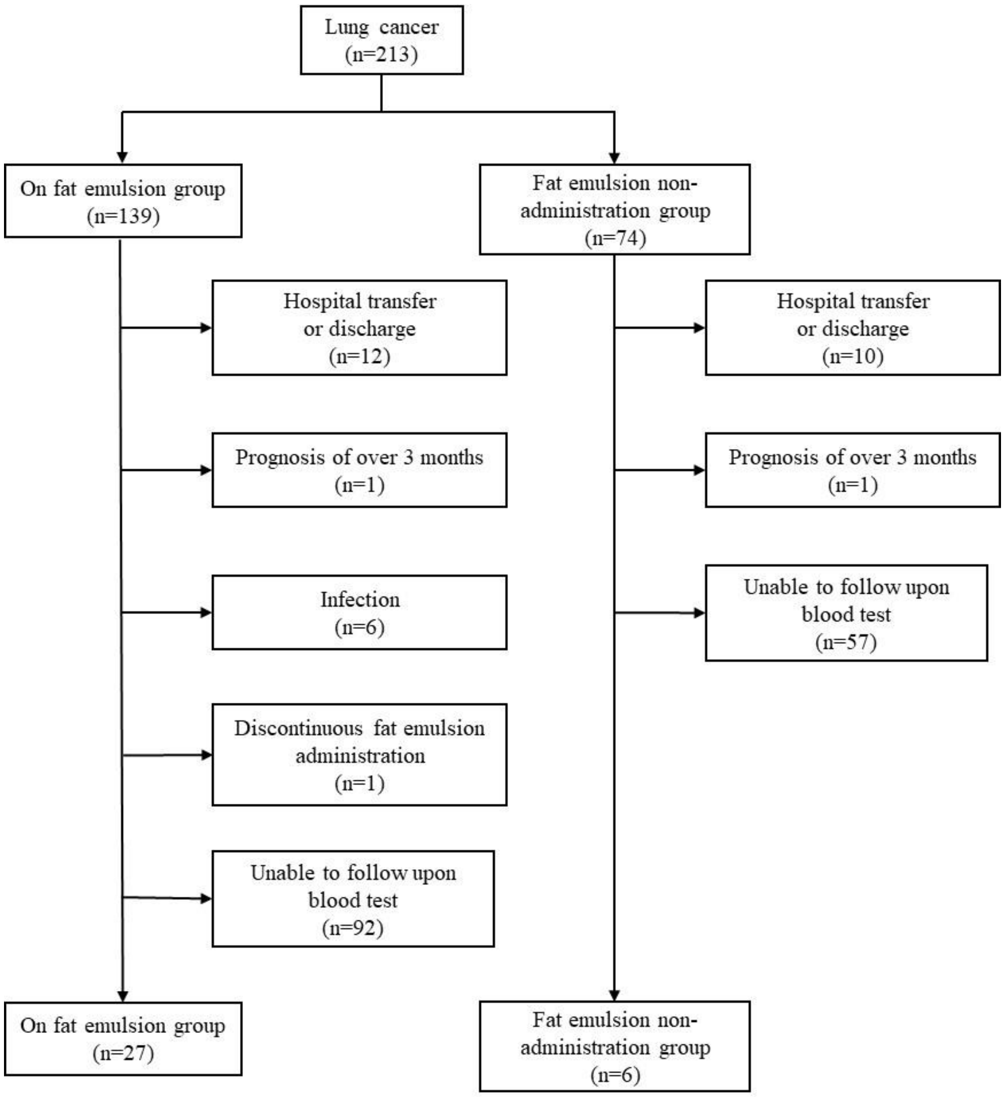

Figure 1. Patients selection flowchart

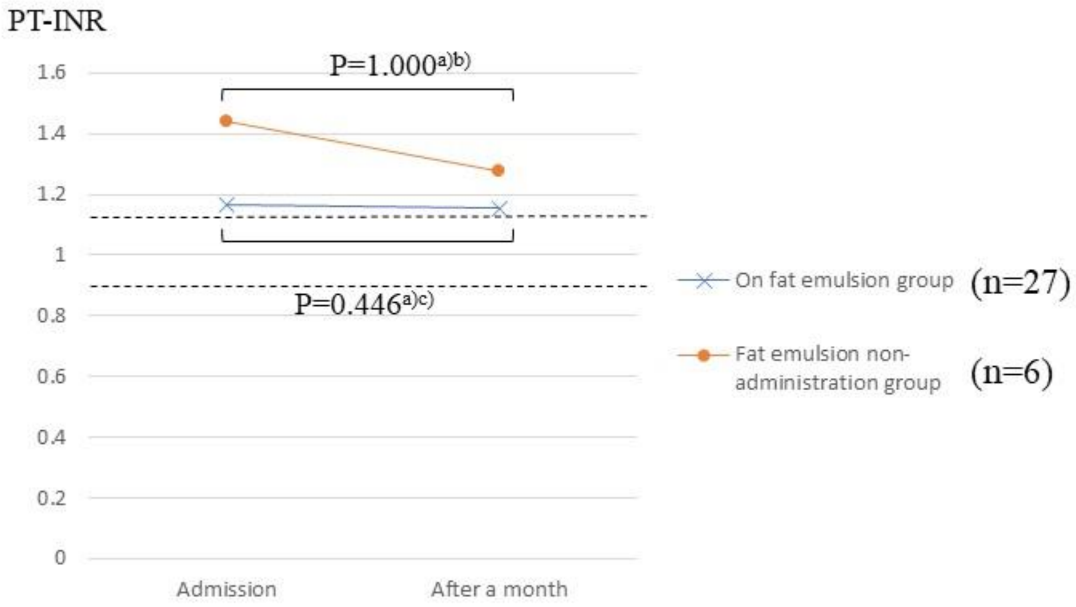

Figure 2. Comparison of PT-INR between the two groups

The values of PT-INR (SD: standard deviation) in on fat emulsion group were 1.17±0.26 (admission) and 1.16±0.12 (after a month). The values of PT-INR (SD) in fat emulsion non-administration group were 1.44±0.43 (admission) and 1.28±0.18 (after a month).

a) Wilcoxon signed-rank test, b) 1-β=0.174, c) 1-β=0.0405

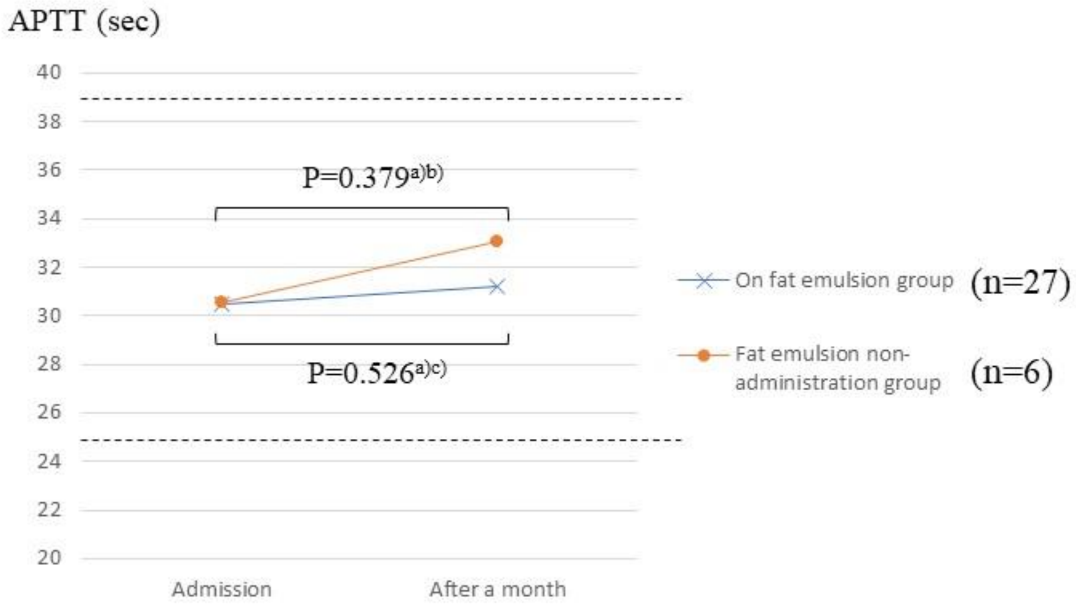

Figure 3. Comparison of APTT between the two groups

The values of APTT (SD) in on fat emulsion group were 30.5±5.0 (admission) and 31.2±4.2 (after a month). The values of APTT (SD) in fat emulsion non-administration group were 30.6±5.2 (admission) and 33.0±7.5 (after a month).

a) Paired t test, b) 1-β=0.121, c) 1-β=0.132

Table 1. Patient characteristics (physical examination findings)

	On fat emulsion group (n=139)	Fat emulsion non-administration group (n=74)	p-value
Age (years)	77.6 ± 9.2	77.7 ± 9.9	0.752[a)]
Sex (female)	41	20	0.752[b)]
Height (cm)	159.6 ± 10.1	158.9 ± 10.3	0.666[a)]
Weight (kg)	48.6 ± 10.3	49.7 ± 12.0	0.724[a)]
BMI (kg/m^2)	19.0 ± 3.3	19.6 ± 4.2	0.664[a)]
Lung cancer			
Small-cell	14	7	
Non-small cell	82	40	0.716[b)]
Unknown	43	27	
Survival time			
Median (day)	25	16.5	0.135[a)]
Performance status			
2	2	1	
3	44	32	0.203[b)]
4	92	41	
Metastasis			
Liver	27	18	
Bone	44	19	
Peritoneum	8	4	0.766[b)]
Brain	47	19	
Lymph node	30	16	

Data is showed by Mean ± SD

a) Mann–Whitney U test

b) Fisher's exact test

Table 2 . Patient characteristics (blood investigation findings)

	Normal range	On fat emulsion group (n=139)	Fat emulsion non-administration group (n=74)	p-value
PT-INR	0.86-1.09	1.25 ± 0.4	1.27 ± 0.3	0.514[a)]
APTT (sec)	24-39	32.9 ± 6.5	33.0 ± 7.1	0.921[a)]
ALB (g/dL)	4.1-5.1	2.8 ± 0.7	2.6 ± 0.6	0.017[b)]
TTR (mg/dL)	22-40	13.4 ± 7.5	12.0 ± 6.6	0.296[a)]
CRP (mg/dL)	0-0.14	6.5 ± 7.3	7.4 ± 7.4	0.245[a)]
TC (mg/dL)	124-222	173.2 ± 51.3	168.9 ± 44.5	0.6[a)]
TG (mg/dL)	30-149	115.5 ± 54.3	121.8 ± 57.8	0.48[a)]
AST (IU/L)	13-20	43.1 ± 65.5	46.3 ± 55.9	0.877[a)]
ALT (IU/L)	10-42	32.5 ± 43.3	32.6 ± 42.4	0.688[a)]

Data is showed by Mean ± SD

a) Mann–Whitney U test

b) t test

Table 3. Underlying medical conditions and characteristics of analyzed patients (physical examination findings)

	On fat emulsion group (n=27)	Fat emulsion non-administration group (n=6)	p-value
Age (years)	77.0 ± 8.5	76.4 ± 4.2	0.907a)
Sex (female)	11	1	0.379b)
Height (cm)	159.1 ± 9.4	160.0 ± 10.3	0.657a)
Weight (kg)	47.7 ± 10.7	48.9 ± 11.9	0.815a)
BMI (kg/m^2)	18.1 ± 4.9	18.9 ± 3.0	0.640a)
Lung cancer			
Small-cell	2	1	
Non-small cell	19	3	0.466b)
Unknown	6	2	
Survival time			
Median (day)	54	67.5	0.207a)
Performance status			
2	2	1	
3	16	5	0.264b)
4	9	0	
Metastasis			
Liver	8	0	
Bone	8	0	
Peritoneum	2	1	0.463b)
Brain	12	1	
Lymph node	9	1	

Data is showed by Mean ± SD

a) Mann–Whitney U test

b) Fisher's exact test

Table 4. Underlying medical conditions and characteristics of analyzed patients (blood investigation findings)

	Normal range	On fat emulsion group (n=27)	Fat emulsion non-administration group (n=6)	p-value
PT-INR	0.86-1.09	1.17 ± 0.26	1.44 ± 0.43	0.0161[a)]
APTT (sec)	24-39	30.5 ± 5.0	30.6 ± 5.2	0.779[a)]
ALB (g/dL)	4.1-5.1	3.1 ± 0.6	2.8 ± 0.3	0.23[b)]
TTR (mg/dL)	22-40	16.5 ± 9.0	17.5 ± 8.6	0.691[a)]
CRP (mg/dL)	0-0.14	4.19 ± 4.76	3.98 ± 5.05	0.815[a)]
TC (mg/dL)	124-222	182.5 ± 41.4	162.5 ± 38.1	0.287[b)]
TG (mg/dL)	30-149	118.4 ± 43.2	105.2 ± 41.4	0.726[a)]
AST (IU/L)	13-20	27.9 ± 13.0	33.7 ± 20.3	0.513[a)]
ALT (IU/L)	10-42	26.1 ± 24.6	27.8 ± 23.9	0.87[a)]
WBC ($\times 10^3/\mu g$)	3.3-8.6	9. 1 ± 4.2	7.8 ± 3.0	0.469[a)]
RBC ($\times 10^6/\mu g$)	3.86-4.92	4.01 ± 0.66	3.68 ± 0.61	0.268[b)]
PLT ($\times 10^4/\mu g$)	15.8-34.8	31.2 ± 12.4	25.1 ± 6.8	0.259[b)]

Data is showed by Mean ± SD

a) Mann–Whitney U test

b) t test

Table 5. Impact on items in On fat emulsion group (n=27)

	Normal range	admission	after a month	p-value
ALB (g/dL)	4.1-5.1	3.1 ± 0.6	2.5 ± 0.5	<0.05[b)]
TTR (mg/dL)	22-40	16.5 ± 9.0	12.7 ± 6.7	<0.05[a)]
CRP (mg/dL)	0-0.14	4.19 ± 4.76	7.40 ± 5.77	<0.05[a)]
TC (mg/dL)	124-222	182.5 ± 41.4	158.2 ± 46.4	<0.05[b)]
TG (mg/dL)	30-149	118.4 ± 43.2	113.5 ± 54.7	0.602[b)]
AST (IU/L)	13-20	27.9 ± 13.0	36.8 ± 23.7	<0.05[a)]
ALT (IU/L)	10-42	26.1 ± 24.6	30.8 ± 22.4	0.115[a)]
WBC ($\times 10^3$/μg)	3.3-8.6	9. 1 ± 4.2	11.5 ± 5.5	<0.05[a)]
RBC ($\times 10^6$/μg)	3.86-4.92	4.01 ± 0.66	3.68 ± 0.73	<0.05[b)]
PLT ($\times 10^4$/μg)	15.8-34.8	31.2 ± 12.4	30.4 ± 12.1	0.739[b)]

Data is showed by Mean ± SD

a) Wilcoxon signed-rank test

b) Paired t test

Table 6. Impact on items in fat emulsion non-administration group (n=6)

	Normal range	admission	after a month	p-value
ALB (g/dL)	4.1-5.1	2.8 ± 0.3	2.6 ± 0.6	0.235[b)]
TTR (mg/dL)	22-40	17.5 ± 8.6	14.6 ± 10.9	0.128[b)]
CRP (mg/dL)	0-0.14	3.98 ± 5.05	5.95 ± 4.31	0.172[b)]
TC (mg/dL)	124-222	162.5 ± 38.1	161.5 ± 53.0	0.918[b)]
TG (mg/dL)	30-149	105.2 ± 41.4	102.3 ± 20.2	0.777[b)]
AST (IU/L)	13-20	33.7 ± 20.3	22.2 ± 6.0	0.438[a)]
ALT (IU/L)	10-42	27.8 ± 23.9	12.5 ± 6.6	0.136[a)]
WBC ($\times 10^3$/μg)	3.3-8.6	7.8 ± 3.0	7.3 ± 1.1	0.662[b)]
RBC ($\times 10^6$/μg)	3.86-4.92	3.68 ± 0.61	3.78 ± 0.75	0.435[b)]
PLT ($\times 10^4$/μg)	15.8-34.8	25.1 ± 6.8	26.6 ± 10.7	0.508[b)]

Data is showed by Mean ± SD

a) Wilcoxon signed-rank test

b) Paired t test
